# Assessing habitat quality at Poyang Lake based on InVEST and Geodetector modeling

**DOI:** 10.1002/ece3.10759

**Published:** 2023-12-03

**Authors:** Wenrui Yuan, Lingkang Chen, Haixia Chen, Shaofu Deng, Hong Ji, Fenshuo Liang

**Affiliations:** ^1^ College of Sciences Guangdong University of Petrochemical Technology Maoming China; ^2^ School of Resource and Environmental Engineering Jiangxi University of Science and Technology Ganzhou China; ^3^ College of Petroleum Engineering Guangdong University of Petrochemical Technology Maoming China

**Keywords:** artificial activity, Geodetectors, Poyang Lake, spatial heterogeneity, wetland ecological environment

## Abstract

Poyang Lake is an essential natural wetland in the Yangtze River basin and plays a vital role in maintaining the ecosystem function and ecological security in the middle and lower reaches of the Yangtze River. However, the relative importance and spatial heterogeneity of the impacts of human activities and land use changes on ecological security needs to be further explored. Here, we analyzed the habitat quality level around Poyang Lake in 2022 and explored the factors of habitat quality change from a geographical perspective. The land use structure changes around the Poyang Lake basin from 2000 to 2022 were quantitatively analyzed, and then the relative importance and spatial heterogeneity of each factor on ecological security changes were investigated using geographic probes. The results show that (1) The worst quality habitat (0–0.1) consists mainly of construction land (1624.9 km^2^) with an area of 1634.64 km^2^; (2) Construction land continues to increase with the most significant change, and the dynamic land use attitude is 0.47. Grassland and mudflats have the greatest decrease. The increase in cultivated land in different periods is mainly due to the shift of water surface and forest land; (3) The drivers of habitat quality in Poyang Lake were significantly influenced by the interaction of socioeconomic factors. The explanatory power of population density interacting with the total year‐end population and population density interacting with administrative area exceeded 0.84. These values were higher than the explanatory power of each individual factor, indicating that habitat quality was primarily associated with population density, total year‐end population, and administrative area. These results suggest that human activities contribute to the degradation of wetlands around Poyang Lake. This study has significant reference value for coordinating human–land relationships in Poyang Lake, optimizing land management policy, and improving the sustainable development of cities.

## Graphical abstract

- Human activities has led to wetland degradation around the Poyang Lake area since 2000.
- Wetland land use change drivers are more influenced by socioeconomic factors.
- Optimizing human land relations to promote sustainable development.

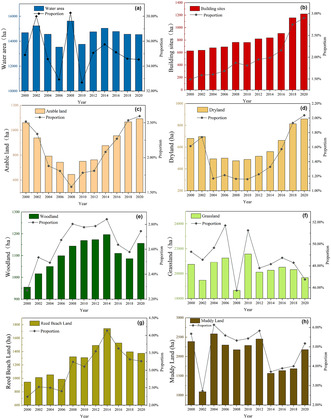

## 1 INTRODUCTION

Wetlands, which evolved over billions of years of soil, water, and life development on Earth, are one of the most biodiverse ecological landscapes in nature and the most important environment for human survival (Song et al., 2021). Wetland systems often have important ecosystem functions such as flood reduction, carbon storage, and biodiversity conservation (Fluet‐Chouinard et al., 2023; Ye et al., 2019). The ecology of many natural wetlands around the world has changed dramatically due to increased human activities over the past century (Awange et al., 2008). Poyang Lake is the largest freshwater lake in China (Han et al., 2015) and the wetlands of Poyang Lake provide the largest winter habitat for more than 90% of Siberian migratory birds (Mei et al., 2016). With increasing urbanization around Poyang Lake (Michishita et al., 2012), the lake's area has been reduced to less than 2000 km^2^ (Han et al., 2015). In the Poyang Lake wetland, the primary threat to nature conservation is the deterioration of habitat quality brought about by land use changes (Yang, Gao, et al., 2020; Yang, Xia, et al., 2020; Zhang, Liao, et al., 2023; Zhang, Wang, et al., 2023). The composition of land use emerges as a significant factor to this, and it also has a substantial impact on the lake's water quality (An et al., 2022; Wang et al., 2023). Therefore, clarifying the relevant impacts of human activities on land use and exploring the drivers of wetland habitat quality change and degradation can help rational development and environmental protection of wetlands.

This study adopted the Integrated Valuation of Ecosystem Services and Trade‐offs (InVEST) habitat quality modeling approach. The InVEST model can quantify the ecosystem's contribution and potential economic value, providing a scientific basis for environmental management and protection. Habitat quality is the ability of an ecosystem to provide sustainable conditions for organisms and is a prerequisite and basis for ecosystem functions and services (Moreira et al., 2018). It reflects the suitability of the environment for human survival, reproduction, and productivity. It is also an important indicator of the ecosystem's health (Tang et al., 2022). Habitat quality indices can be used to characterize habitat quality and evaluate biodiversity levels in the study area. They reflect species' genetic variation and potential during reproduction (Sharp et al., 2018). In past studies, many scholars have successfully applied the InVEST model to assess habitat quality (Qiao et al., 2023; Wang, Ma, et al., 2022; Wang, Ye, et al., 2022; Wei et al., 2022; Zhang, Liao, et al., 2023; Zhang, Wang, et al., 2023).

The level of habitat degradation is a crucial indicator of the ecological health of Poyang Lake (Xu et al., 2022). Habitat degradation can manifest in several ways, including water quality pollution, lakeshore zone degradation, and eutrophication of lake waters (Feng et al., 2016; Li et al., 2020, 2021). Studies have indicated (Lu et al., 2011) that these degradation phenomena have had negative impacts on the aquatic biodiversity, fish resources, and wetland functions of Poyang Lake (Lei et al., 2011; Mu et al., 2020; Yang et al., 2018). Therefore, the main drivers and their contribution rates need further quantitative studies. In this study, we introduced a model, Geodetector, to comprehensively assess the level of habitat degradation in Poyang Lake. Geoprobe is a novel approach that does not rely on linear assumptions (Hua & Hao, 2021; Wu et al., 2022) and can reveal the degree of contribution of various factors to ecosystem change. This innovative method provides a new way to quantitatively characterize the drivers of wetland habitat degradation.

In this study, we assessed the habitat quality and degradation of Poyang Lake wetlands in 2020 using the InVEST model and quantified the drivers with Geodetector. These assessments were further analyzed in conjunction with land use changes from 2000 to 2020. Additionally, the main drivers affecting habitat quality were quantitatively identified through Geodetector's driver analysis. The research findings will serve as a valuable reference for future management and protection of habitat quality in Poyang Lake.

## 2 MATERIALS AND METHODS

### 2.1 Study areas

**FIGURE 1:**
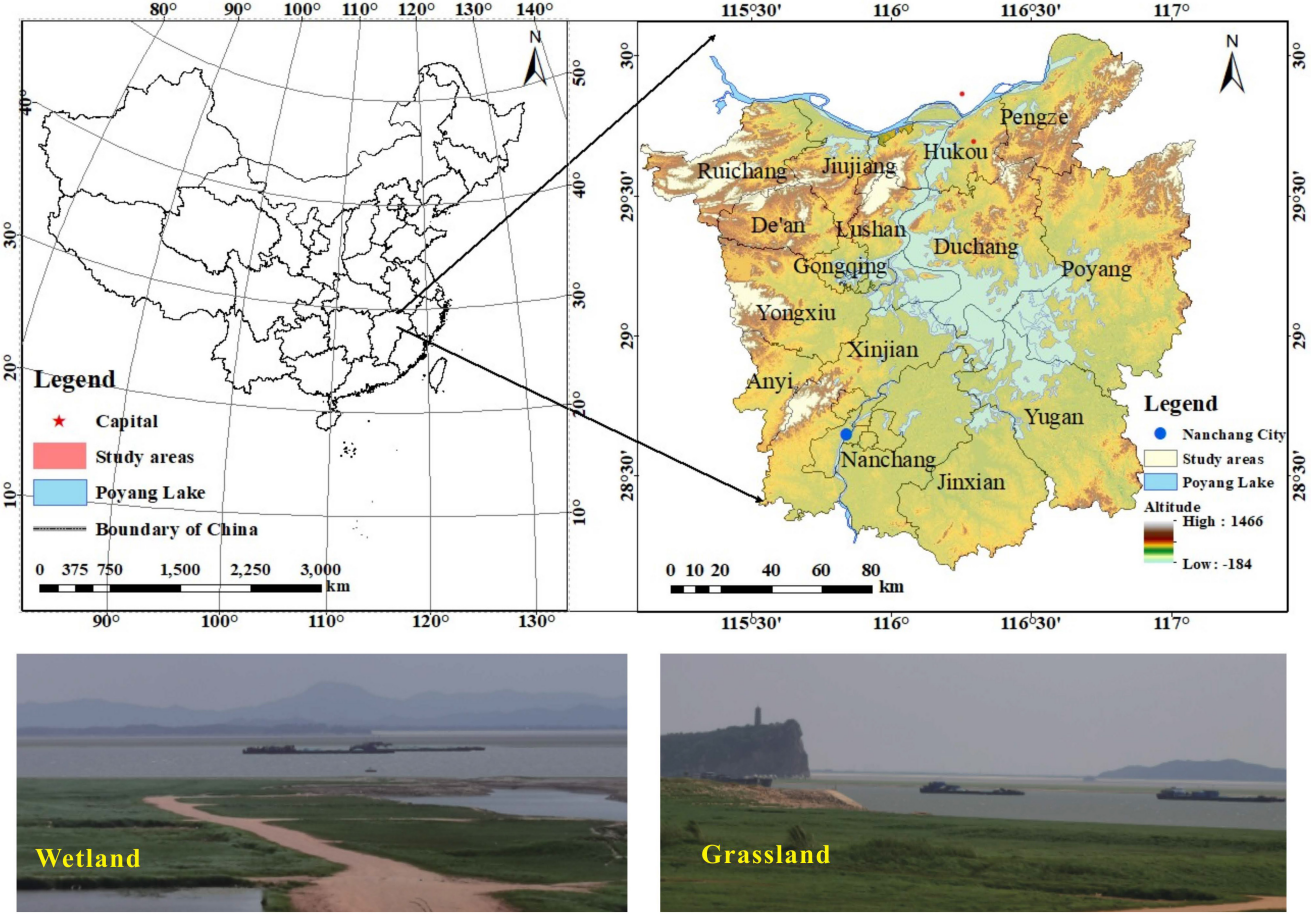
The location of Poyang Lake and the local scenery.

Poyang Lake is located in the north of Jiangxi Province and is the largest freshwater lake in China (Figure 1). It plays a vital role in water storage, irrigation, and flood regulation. The lake is an important throughput shallow lake in the Yangtze River basin (Sjögersten et al., 2014). Poyang Lake National Nature Reserve, located in the northwest corner of Poyang Lake, with geographical coordinates 115°47′ E–116°45′ E, 28°22′ N–29°45′ N, straddles Yongxiu and Xingzi counties and is centered on the town of Wu in Yongxiu County.

The population density of Nanchang City and its surrounding areas in the southwest of Poyang Lake can reach a maximum of 600 persons/km^2^ (Liu & Ye, 2022). The study includes 15 administrative areas in Jiujiang, Ruichang, Hukou, Pengze, De'an, Lushan, Duchang, Gongqingcheng, Yongxiu, Poyang, Anyi, Xinjian, Nanchang, Yugan, Nanchang, and Jinxian counties.

The population expansion of cities and counties around Poyang Lake seriously threatens the wetlands. Reconciling economic and social development with the ecological health of wetlands is a necessary and comprehensive task. Analyzing the main drivers and impacts of wetland change is important to achieving this goal (Wang, Ma, et al., 2022; Wang, Ye, et al., 2022).

### 2.2 Data sources

The data used in this study mainly include Landsat remote sensing images, a digital elevation model, and socioeconomic data of the Poyang Lake area. Landsat remote sensing images were taken from the United States Geological Survey (USGS), 2000–2020, with a spatial resolution of 30 m. Socioeconomic statistics are obtained from the Statistical Yearbook of Jiangxi Province (jiangxi.gov.cn) from 2000 to 2022.

### 2.3 Methods

#### 2.3.1 Evaluation of habitat quality

In this article, the habitat quality module of the InVEST model is used to assess the habitat quality of the study area for the period 2000–2020. Habitat quality is considered a continuous variable, considering the distance and spatial weight of stressors, the degree of legal protection of the land, and the effects of changes in land cover patterns and land cover patterns on habitat quality. The index values range between 0 and 1 (Tang et al., 2020) and are calculated as follows:

(1) $$D_{\mathrm{xj}}=\sum_{r=1}^{R}\sum_{y=1}^{Y_{r}}\left(w_{r}/\sum_{r=1}^{R}w_{r}\right)r_{y}i_{\mathrm{rxy}}\beta_{x}S_{\mathrm{jr}}$$

where *D*_*xj*_ is the degree of habitat degradation of raster *x* in habitat type *j*; *R* is the number of threat sources; *W*_*r*_ is the weight of threat source *r*; *Y*_*r*_ is the number of rasters of the threat element; *r*_*y*_ is the coercion value of raster *y*; *β*_*x*_ is the accessibility of the threat element to raster *x* (the value of *β*_*x*_ is determined between 0 and 1 depending on its level of legal protection); *S*_*jr*_ is the sensitivity of habitat type *j* to threat source *r*; and *i*_*rxy*_ is the coercion value of *r*_*y*_ to raster *x* of raster *y* levels of threat, divided into linear and exponential decay:

(2) $$\text{Linear decay}:i_{\mathrm{rxy}}=1-\left(\frac{d_{\mathrm{xy}}}{d_{r\max}}\right)$$

(3) $$\text{Exponential decay}:i_{\mathrm{rxy}}=\exp\left(\frac{-2.99\mathrm{dxy}}{d_{r\max}}\right)$$

where *d*_*xy*_ is the linear distance between grid *x* and grid *y*; and *d*_*r*max_ is the maximum stress distance of the threat source *r*. Habitat quality is calculated as

(4) $$Q_{\mathrm{xj}}=H_{j}\left[1-\left(\frac{D_{\mathrm{xj}}^{z}}{D_{\mathrm{xj}}^{z}+k^{z}}\right)\right]$$

where *Q*_*xj*_ is the habitat quality index for raster *x* in habitat type *j*; *H*_*j*_ is the habitat suitability of habitat type *j* (0 ≤ *H*_*j*_ ≤ 1); and *k* is the half‐saturation constant, taken as half of the maximum habitat degradation, generally set to 0.5; *z* is the normalization constant, generally taken as 2.5.

In this article, we applied InVEST version 3.12.0 for habitat quality model operation. We carried out a series of processing such as vectorization, reclassification, raster calculation, and data aggregation on the land use cover data of the Poyang Lake watershed in Jiangxi Province in the ArcMap 10.8 platform, assigning a value of 1 to selected land types and 0 to the rest. There are two key issues when running the InVEST model to assess habitat quality. The first is the selection of stressors. Poyang Lake flows through Nanchang, an important transportation hub in Jiangxi Province in the middle and lower reaches of the Yangtze River in China. The expansion of construction land and human activities are the main threats to habitat patches in the context of rapid urbanization and industrialization. Based on the actual situation of Poyang Lake in Jiangxi Province, five types of land, namely Paddy field, Dryland, Urban land, Rural residential land, and Industrial and traffic land, were extracted as threat sources (Moreira et al., 2018; Tang et al., 2022). The five land types, Dryland, Urban land, Rural residential land, and Industrial and traffic land, are sources of threat (Moreira et al., 2018; Tang et al., 2022).

Second, the parameters were determined. Model parameters were identified with reference to relevant studies (He et al., 2017; Tang et al., 2020) to determine the relevant maximum stress distances, weights, and attenuation types (Table 1). The remaining habitat suitability and sensitivity to stressors for different types are shown in Table 2.

**TABLE 1:** Attributes of the stressors.

Stressors	Maximum distance	Weight	Spatial decay type
Paddy field	6	0.6	Exponential
Dry land	6	0.6	Exponential
Urban land	10	0.9	Exponential
Rural residential land	8	0.7	Exponential
Industrial and traffic land	12	1	Linear

**TABLE 2:** Habitat suitability and sensitivity of land use type to each stressor.

Land use type	Habitat suitability	Paddy field	Dry land	Urban land	Rural residential land	Industrial and traffic land
Paddy	0.3	0	0.2	0.6	0.4	0.5
Dry land	0.3	0.2	0	0.6	0.4	0.5
Forestland	l	0.8	0.8	0.9	0.8	0.9
Shrubland	0.9	0.5	0.5	0.8	0.7	0.8
Grassland	0.8	0.5	0.5	0.6	0.5	0.6
Water body	1	0.8	0.7	0.9	0.8	0.9
Urban	0	0	0	0	0	0
Bare land	0.1	0.1	0.1	0.2	0.2	0.1

#### 2.3.2 Land use

The total area assessed by the model is 24,715 km^2^ and includes the seven different land use types being studied. The agricultural land surrounding Poyang Lake corresponds to 50.6% (12,524.3 km^2^) of the total area assessed and is mainly found in the plains on the periphery of the Poyang Lake water body. The water bodies of Poyang Lake account for 15% (3720 km^2^) of the total assessed area, and the forests account for 27.6% (6829 km^2^) of the total assessed area. Grassland, submerged vegetation (6.25 km^2^), bare ground (9.74 km^2^), shrubs (0.07 km^2^), and building land (1624.9 km^2^) together account for 6.6% of all habitats assessed.

This article combines the remote sensing monitoring database of China's land use status in 2000, 2010, and 2020. It uses the single and the integrated land use type dynamic attitude to conduct the analysis.

(1) Single land use type dynamic attitude.

The single land use type dynamic attitude refers to the change in a land use type in the study area over a certain period. It is used to characterize the spatial and temporal changes in different land use types over a certain period. The expression is (Wang & Bao, 1999):

(5) $$K_{i}=\frac{U_{\mathrm{ib}}-U_{\mathrm{ia}}}{U_{\mathrm{ia}}}\times \frac{1}{T_{b}-T_{a}}\times 100\%$$

where *K*_*i*_ is the dynamic attitude of land use type *i* in period a ~ b; *Ui*_*a*_ and *Ui*_*b*_ are the areas of land type_*i*_ at times *a* and *b*, respectively (hm^2^); and *T*_*a*_ and *T*_*b*_ refer to the early stage of the study *a* and the end of study *b*, respectively.

A higher value of *K*_*i*_ indicates a more significant conversion from other types of land to this type of land and a more significant relative change over the study period; conversely, a lower value of *K*_*i*_ indicates a smaller change.

(2) Integrated land use types move attitudes.

The integrated land use dynamic attitude indicates the land use change rate in the study area over a certain period. The specific expression is (Wang & Bao, 1999):

(6) $$\mathrm{LC}=\frac{\sum_{i=1}^{n}\;\left(\Delta LU_{i-j}\right)}{2\times \sum_{i=1}^{n}\;LU_{i}}\times \frac{1}{T}\times 100\%$$

where *LC* denotes the combined land use dynamics in the study area; n is the number of land use types in the study area; *ΔLU*_*i–j*_ is the area of the study area in the study period where type *i* land use is converted to a non‐type i land use type; *LU*_*i*_ is the initial area of type *i* land use in the study area; and *T* is the length of the study period.

A higher *K* value indicates a more significant change in land use in the region; conversely, a lower *K* value indicates a smaller change.

(3) Land use transfer matrix.

To see the transfer between land use types more intuitively and understand the trend and direction of land use change, the land use transfer moment was applied to analyze the land use change in Poyang Lake wetland and visualize the area change in each land use type in Poyang Lake wetland using 20 years of data. The land use transfer matrix was mainly used to analyze the rate and direction of transfer between land use types in different periods and the inner correlation and change trend between land use types. The transfer matrix equation is as follows (Liu & Zhu, 2010):

(7) $$P_{\mathrm{ij}}=\left[\begin{array}{cccc} P11 & P12 & \cdots & P1n\\ P21 & P22 & \vdots & P2n\\ \vdots & \vdots & \ddots & \vdots \\ \mathrm{Pn}1 & \mathrm{Pn}2 & \cdots & \mathrm{Pnn} \end{array}\right]$$

where *P* represents the area of land use type transfer; *n* represents the number of land use types classified in the study area; *i*, *j* (*i*, *j* are integers of 1, 2, 3…. *n* integers) denote the area before and after the transfer of a land use type; and *P*_*ij*_ denotes the number of land use types in category *i* that have been transferred to category *j*.

The land use data of different periods were fused and overlaid by ArcGIS software to obtain two periods of land use transfer matrices. In this study, the 22 years from 2002 to 2022 were divided into two time periods, 2002–2012 and 2012–2022, and the land use transfer matrices for the two periods were established.

Land use change results from the combined effect of physical and socioeconomic constraints (Zhu et al., 2022). The topography and geomorphology of the Poyang Lake basin are the background environmental constraints on spatial and temporal land use changes. The geographical differentiation characteristics of natural elements such as elevation, slope, slope direction, and temperature control the land use type changes in the area (Shi et al., 2023). In the context of economic construction, the urban fringe of the study area has expanded, the industrial structure has been optimized, and uneven urban development differences still objectively exist (Chen et al., 2022). Based on the results of theoretical and empirical analyses of existing research on land use change factors and considering the availability of socioeconomic data and the quantitative nature of natural factors in the study area, nine influencing factors were selected from the topography, climatic conditions, soil type, population distribution and structure, and economic level elements, with factors and descriptions (Table 3).

**TABLE 3:** Impact factors and description of land use change.

Type	Key elements	Factor	Description
Physical Geography	Terrain	X1: Elevation	Elevation of the study area (m)
		X2: Slope	Slope of the study area (°)
		X3: Slope direction	Slope orientation of the study area
	Climatic conditions	X4: Precipitation	Average annual precipitation in the study area (mm)
		X5: Temperature	Average annual temperature in the study area (°C)
Socio‐economic		X6: Population density	Population distribution in the study area (persons/km^2^)
		X7: Total population at the end of the year	Total population of the study area at the end of the year (million)
		X8: Gross domestic product	Study area GDP (million)
		X9: Area of the district	Area of the administrative area of the study region (km^2^)

#### 2.3.3 Geodetectors

Geodetector is a saliency method oriented toward spatial data that reveal the influence of driving variables on geographical phenomena and detect heterogeneity of geographical elements of the same criteria in spatial layer clouds. By detecting the spatial analytic properties of regions through Geodetector, we can reflect the similarity of physical phenomena in the same region and the difference between different regions and then analyze the driving forces that form the spatial variation of geographical phenomena (Hua & Hao, 2021; Wu et al., 2022). As a result, it has been widely used in ecology (Hu et al., 2020), public health (Zhang, Xu, & Xiao, 2020; Zhang, Zhou, & Li, 2020), regional economics (Wang & Hu, 2012), and other fields (Zhang, Xu, & Xiao, 2020; Zhang, Zhou, & Li, 2020).

Factor detection is used to detect the spatial heterogeneity of *y* and the extent to which factor *x* explains the spatial heterogeneity of attribute *y*. Factor detection is used to investigate the extent to which factor *x* explains the dependent variable *y*, measured by the value of *q* (Wall et al., 2021). The *q* value presented in the figure represents the amount of independent variable explanation on the dependent variable at the 95% confidence level, and it indicates that all factors for each period passed the hypothesis test at the 5% level.

The expression is as follows (Wang & Hu, 2012):

(8) $$q=1-\frac{\sum_{h=1}^{L}\;N_{h}\sigma_{h}^{2}}{N\sigma^{2}}=1-\frac{\mathrm{SSW}}{\mathrm{SST}}$$

(9) $$\mathrm{SSW}=\sum_{h=1}^{L}\;N_{h}o_{h}^{2}$$

(10) $$\mathrm{SST}=N\sigma^{2}$$

where 𝑇𝑇𝑈 is the Total Sum of Squares for the whole area, 𝑇𝑇𝑊 is the Within Sum of Squares, *q* is the degree of interpretation of factor *x* to the dependent variable *y*, the value range is 0–1, the larger the value, the more pronounced the spatial heterogeneity, and vice versa.

Interaction detection is used to analyze the interaction between different risk factors and whether two or more factors, when acting together, can increase or decrease the degree of explanation of the dependent variable. A two‐by‐two comparison between the factors makes the determination.

Ecological probes were used to compare whether the effects of the two factors x_1_ and x_2_ on the spatial distribution of attribute y were significantly different, as measured by the F‐statistic:

(11) $$F=\frac{N_{x_{1}}\left(N_{x_{1}}-1\right)\mathrm{SS}W_{x_{1}}}{N_{x_{2}}\left(N_{x_{2}}-1\right)\mathrm{SS}W_{x_{2}}}$$

(12) $$\mathrm{SS}W_{x_{1}}=\sum_{h=1}^{l_{1}}\;N_{h}\sigma_{h}^{2},\mathrm{SS}W_{x_{2}}=\sum_{h=1}^{l_{2}}\;N_{h}\sigma_{h}^{2}$$

where 𝑁_*x*1_, 𝑁_*x*2_ denote the number of samples for factors *x*_1_, *x*_2_, respectively; 𝑇𝑇𝑊_*x*1_, 𝑇𝑇𝑊_*x*2_ denote the sum of intra‐stratum variance. 𝑙_1_ and 𝑙_2_ denote the number of stratifications of samples with factors *x*_1_ and *x*_2_, respectively. The null hypothesis 𝐻0 is that 𝑇𝑇𝑊_*x*1_ = 𝑇𝑇𝑊_*x*2_, and if α rejects the original hypothesis at a significant level, it means that *x*_1_ and *x*_2_ have a significant difference in their effect on the spatial distribution of the dependent variable.

## 3 RESULTS

### 3.1 Habitat quality

The model output maps of habitat quality and relative degradation, which offered qualitative analysis on a 0–1 scale, and these maps were classified into 10 equal numerical classes in our study, with an additional class to represent the highest quality class that could be achieved (1). The watershed habitat quality was classified into five groups based on a quantitative examination of the number of square cells in each class, expressed in square kilometers: excellent (0.8–1.0), good (0.7–0.8), fair (0.5–0.7), poor (0.1–0.5), and awful (0–0.1).

The five categories of habitat degradation are as follows: mill (0–0.01), low (0.01–0.05), rate (0.05–0.10), high (0.10–0.15), and severe (0.15–0.20). Table 4 displays the area and proportion of each type of habitat quality and habitat deterioration.

**TABLE 4:** Proportion and index of habitat quality and habitat degradation area at different levels in Poyang Lake basin.

Grade	Excellent	Good	General	Poor	Difference
Habitat quality index Area share (%)	0.8–1.0	0.7–0.8	0.5–0.7	0.1–0.5	0–0.1
	16.56	13.19	1.23	61.37	7.65
Habitat degradation Index of area (%)	0–0.01	0.01–0.05	0.05–0.10	0.10–0.15	0.15–0.20
	3.26	10.51	78.32	6.36	1.55

#### 3.1.1 Habitat quality levels

**FIGURE 2:**
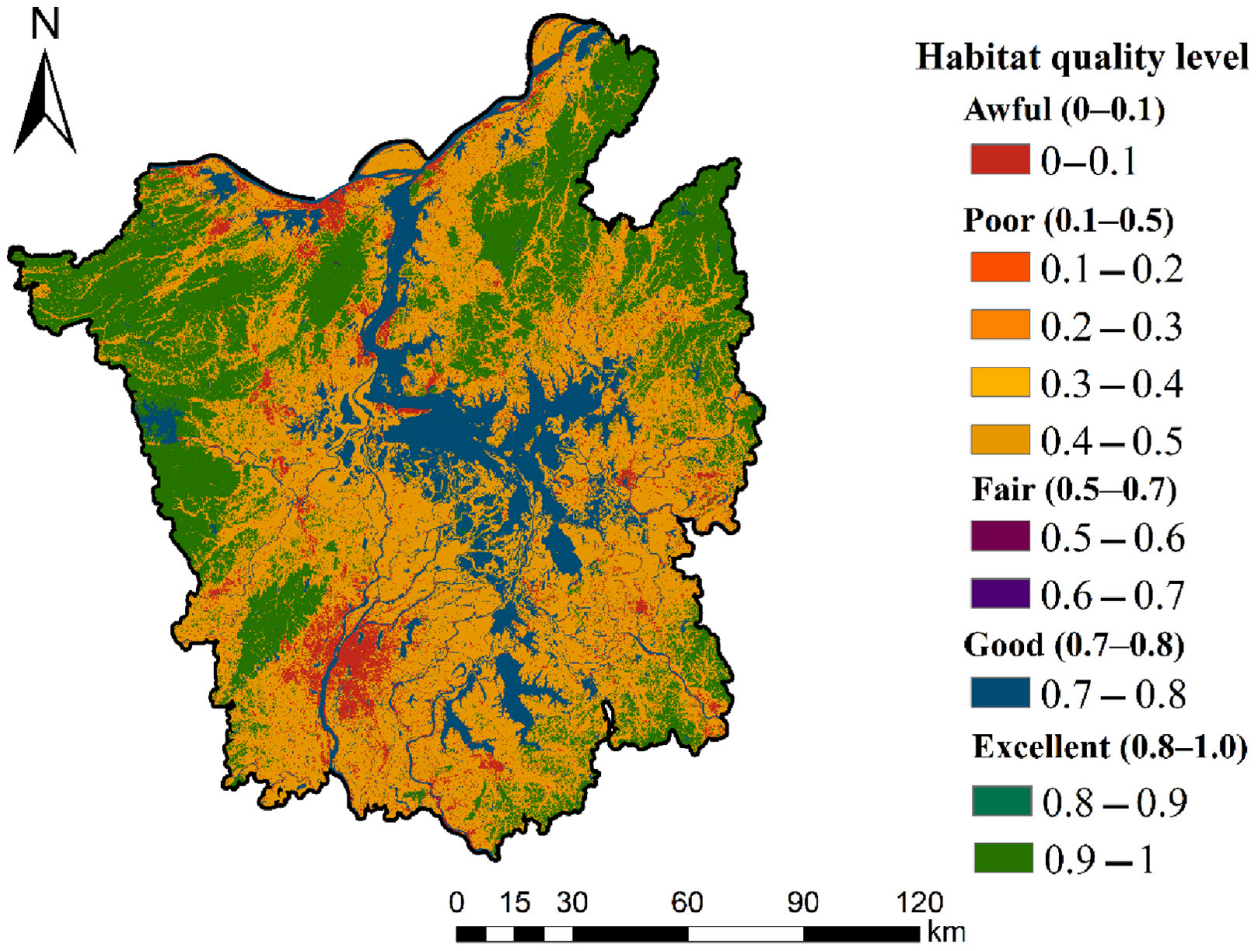
Habitat quality classes around Poyang Lake, 1 being the highest class.

The maps obtained in this study show the values for all quality levels (Figure 2; Table 2). The lowest habitat quality (0–0.1) has 1634.64 km^2^ and consists primarily of built‐up land (1624.9 km^2^). Most are located in urban‐based areas, including Jiujiang and Nanchang, with a continuing trend toward outward expansion (Chen et al., 2020).

As the economic development zone in Nanchang began to expand around 2003 (Yan et al., 2013), Nanchang County expanded to the west and north from 2004 to 2009 following the onset of widespread urbanization. In addition, the economic development plan issued by the Jiangxi Provincial Development and Reform Commission (http://drc.jiangxi.gov.cn/) has led to rapid urbanization in Nanchang and surrounding counties. The counties in the study are all distributed in small pockets, and the habitat quality around building sites may correlate with their size.

The highest habitat quality levels (0.9–1) exist around Mount Lushan and some hills around Poyang Lake, with 98% of the habitat area in the Mount Lushan region showing the highest quality levels. The highest quality levels represent habitats without any threats. This fact is explained by the altitude of this natural habitat, which is 1000 m above sea level on Mount Lushan, where human habitats are considered the most significant threat. Tourism can have a negative impact on the habitat (Yang et al., 2020).

In addition, native scrub habitat quality is located in the first three categories, distributed around the mudflats around Poyang Lake, with wetland cover types exhibiting vegetation, mudflats, and water bodies along an elevation gradient from the boundary to the center of the Poyang Lake water body. During the dry season, the vegetation type is mainly meadows (Sheng et al., 2011), distributed in the southern and eastern parts of Poyang Lake. The water bodies are located in the central, eastern, and southern parts, with a small area of sand distributed northward from the central water body around a narrow channel that empties into the Yangtze River. In near‐lake areas, longer dry seasons may result in lower soil moisture due to increased evaporation, which limits the growth of wetland vegetation in these areas (Mu et al., 2020).

Water bodies are exceptional types, with Poyang Lake water bodies showing a quality rating of 0.7–0.8. The Poyang Lake Ecological Reserve covers an area of 224.4 km^2^, and the wetlands of Poyang Lake are mainly in the basin and plain, with a small area in the mountains. The reserve consists of nine lakes: Beng Lake, Dacha Lake, Dahu Pond, Sha Lake, Changhu Pond, Zhonghu Pond, Xiang Lake, Meixi Lake, and Zhushi Lake, as well as a dozen nearby lakes and grassland. The central water bodies all have a habitat quality of 0.75 or above. Except for the land, the whole area of the highest quality class is inside Poyang Lake (Figure 2). Outside the boundary of Poyang Lake, large areas of agricultural land with the lowest quality levels occur, enclosing the water body. This result can be used to delineate the Poyang Lake area boundary, allowing for management improvements and potentially indicating practical management actions.

**FIGURE 3:**
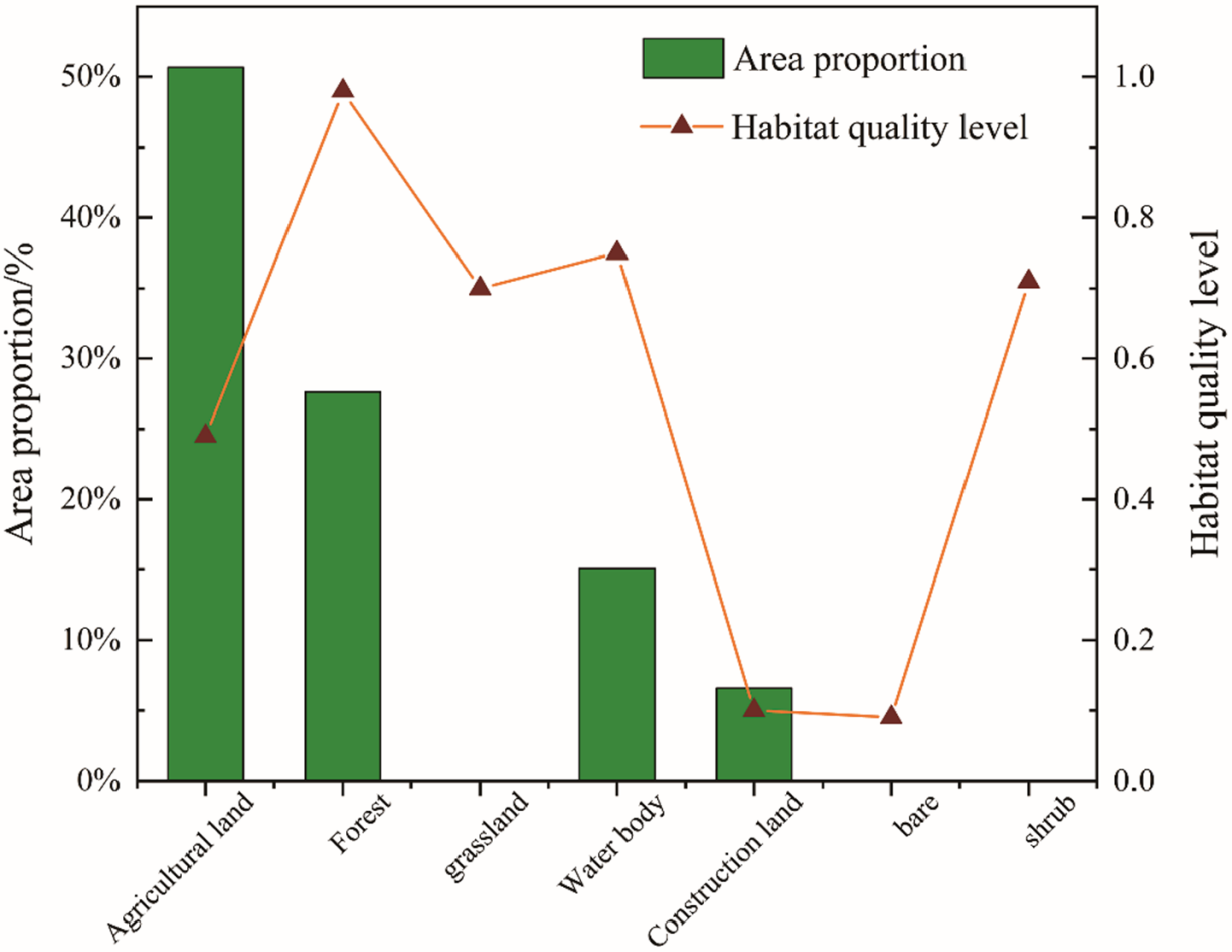
Habitat for different land use types: Proportion of quality habitat area.

Using ArcGIS software, the raster map of habitat degradation was overlaid with the land use map to calculate the distribution of differences in habitat quality by land use type (Figure 3). The reason why grassland, bare ground, and shrubs are not shown is probably because the flooding period of Poyang Lake covered the lower elevation grassland and mudflats and some low shrubs around Poyang Lake (Feng et al., 2012). The above results show that habitat degradation in the watershed is closely related to the watershed. It is evident that various land use types significantly impact habitat degradation.

#### 3.1.2 Habitat degradation level

**FIGURE 4:**
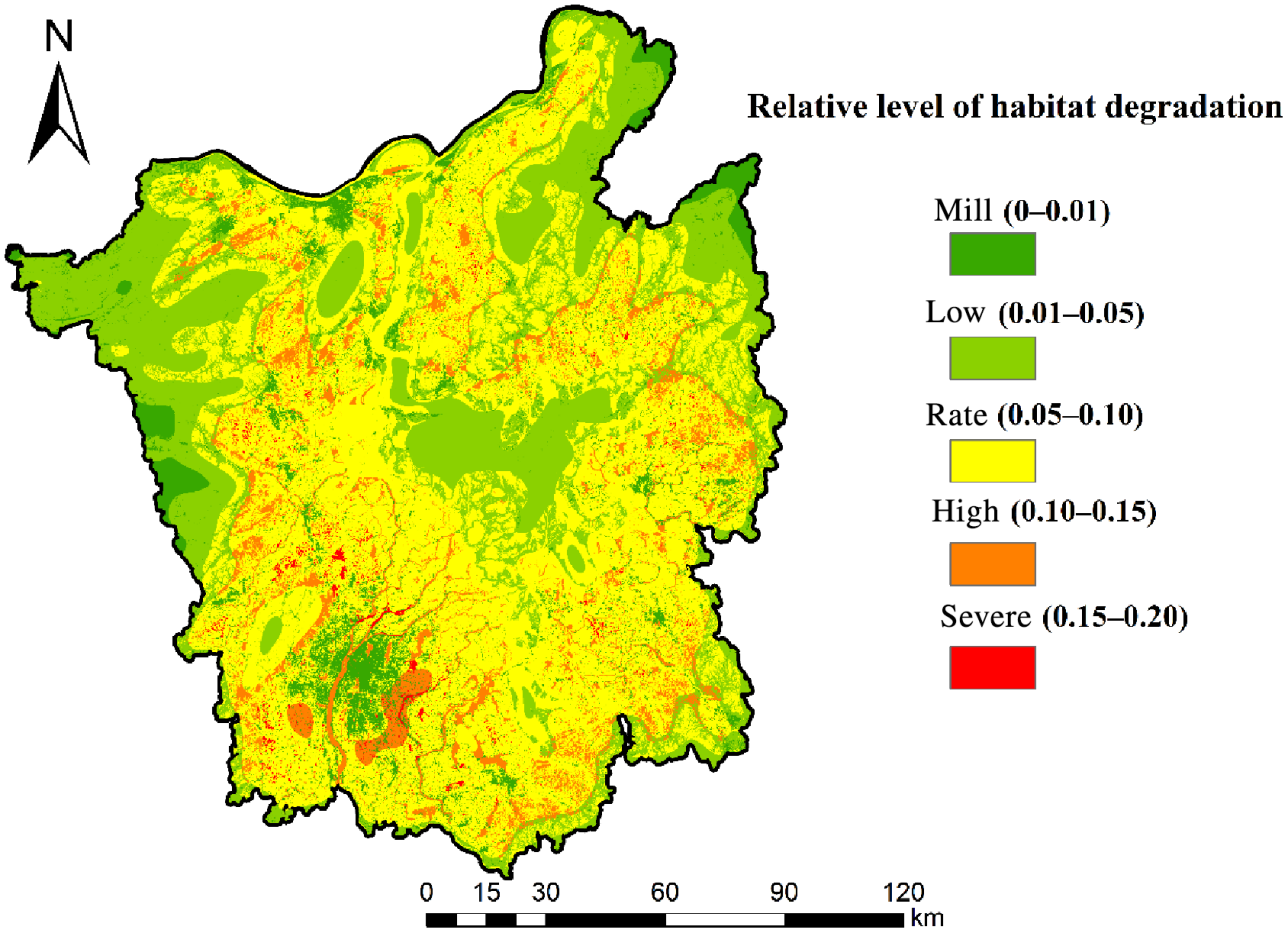
Map showing habitat degradation levels around Poyang Lake, with the highest degradation in the study area at 0.2.

The highest level of degradation reached by the regional habitats in the output map is 0.20 (Figure 4). Since forests and water bodies far from the agricultural land of human activities are less affected, the areas further away from human activity are less threatened; the border of the study area reaches the highest possible quality level.

In this study, the agricultural land around Poyang Lake represents 50.07% of the degradation level (0.05–0.10) area, which is the single habitat reaching the maximum degradation level. The habitat degradation degree of all cities in the Poyang Lake basin has different degrees. The highest habitat degradation degree is in Nanchang City, which has a higher degradation level. The overall habitat degradation in each city shows a decreasing trend and then increasing trend. An integration with land use analysis shows that the level of habitat degradation in the watershed is mainly influenced by woodland, grassland, and cropland. The woodland and grassland habitat suitability is higher, making the water's habitat degradation show a decreasing trend (Michishita et al., 2012).

Analysis of land use change from 2010 to 2018 reveals a significant decrease in the area of forested land with high habitat suitability in the watershed. Conversely, the rapid increase in urbanization in the watershed during this period resulted in a proliferation of construction lands, which exacerbated habitat degradation in the watershed (Dai et al., 2021).

**FIGURE 5:**
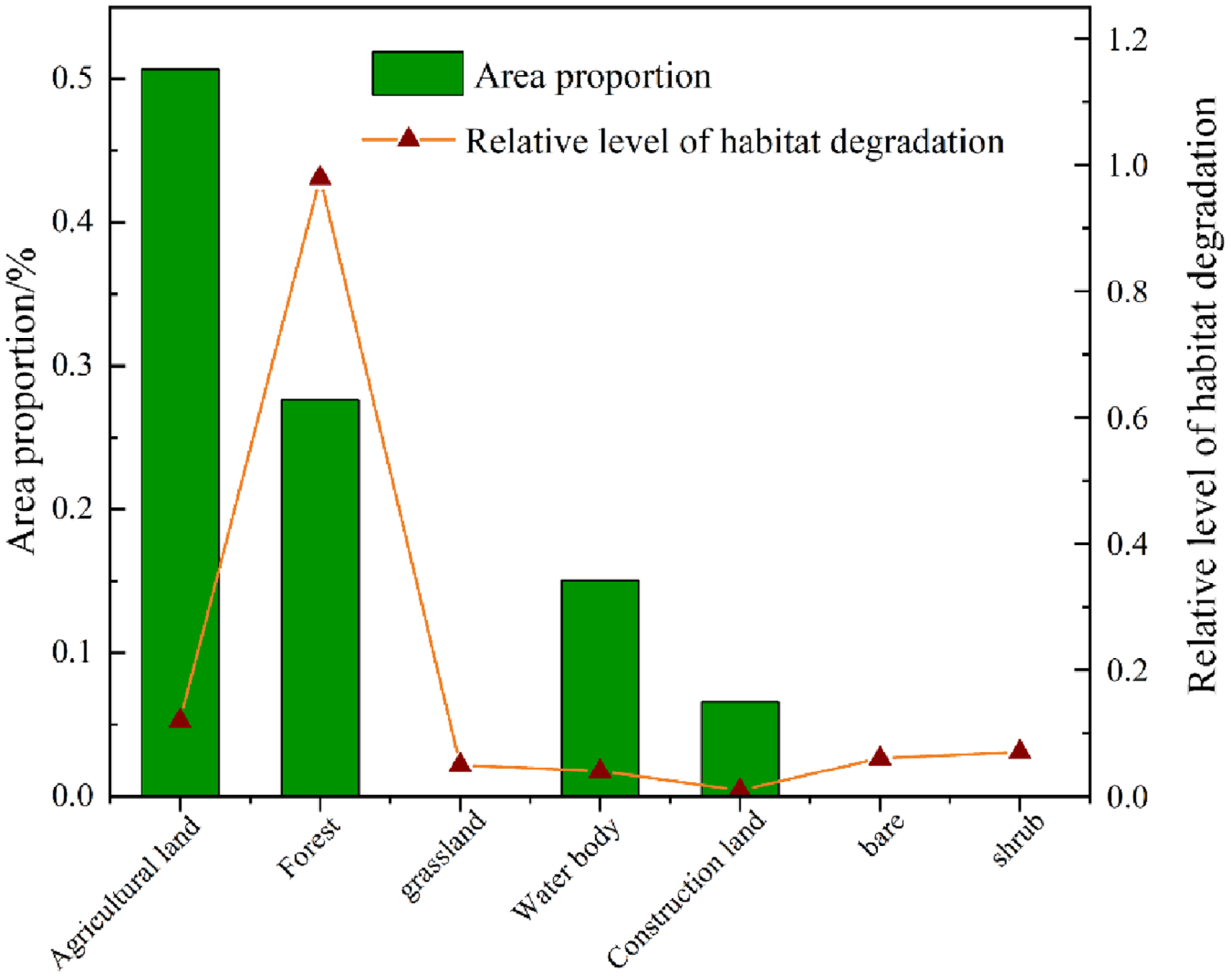
Proportion of habitat degradation area in different land use types.

The raster map of habitat degradation was overlaid with the land use map in ArcGIS to obtain the different distribution of habitat degradation by land use type (Figure 5). Among the various land use types, the habitat degradation degree was in the order of construction land < water body < grassland < bare land < shrub < agricultural land < forest.

Among them, agricultural land accounted for 50.67% of the total watershed area and was the largest land use type with the highest habitat degradation. In comparison, the construction land accounted for 6.57% of the total watershed area and was the highest land use type with 0.01 habitat degradation; bare land and shrub erosion area was the smallest, accounting for only 0.04% of the watershed.

The above results show that habitat degradation in the watershed is closely related to the land use types in the watershed. It can also be found that different land use types have large differences in habitat degradation. The large area of agricultural land with high habitat degradation index has a greater influence on the degradation index of other land types, which has a specific relationship with distance.

### 3.2 Land use

#### 3.2.1 Structural changes in land use

**FIGURE 6:**
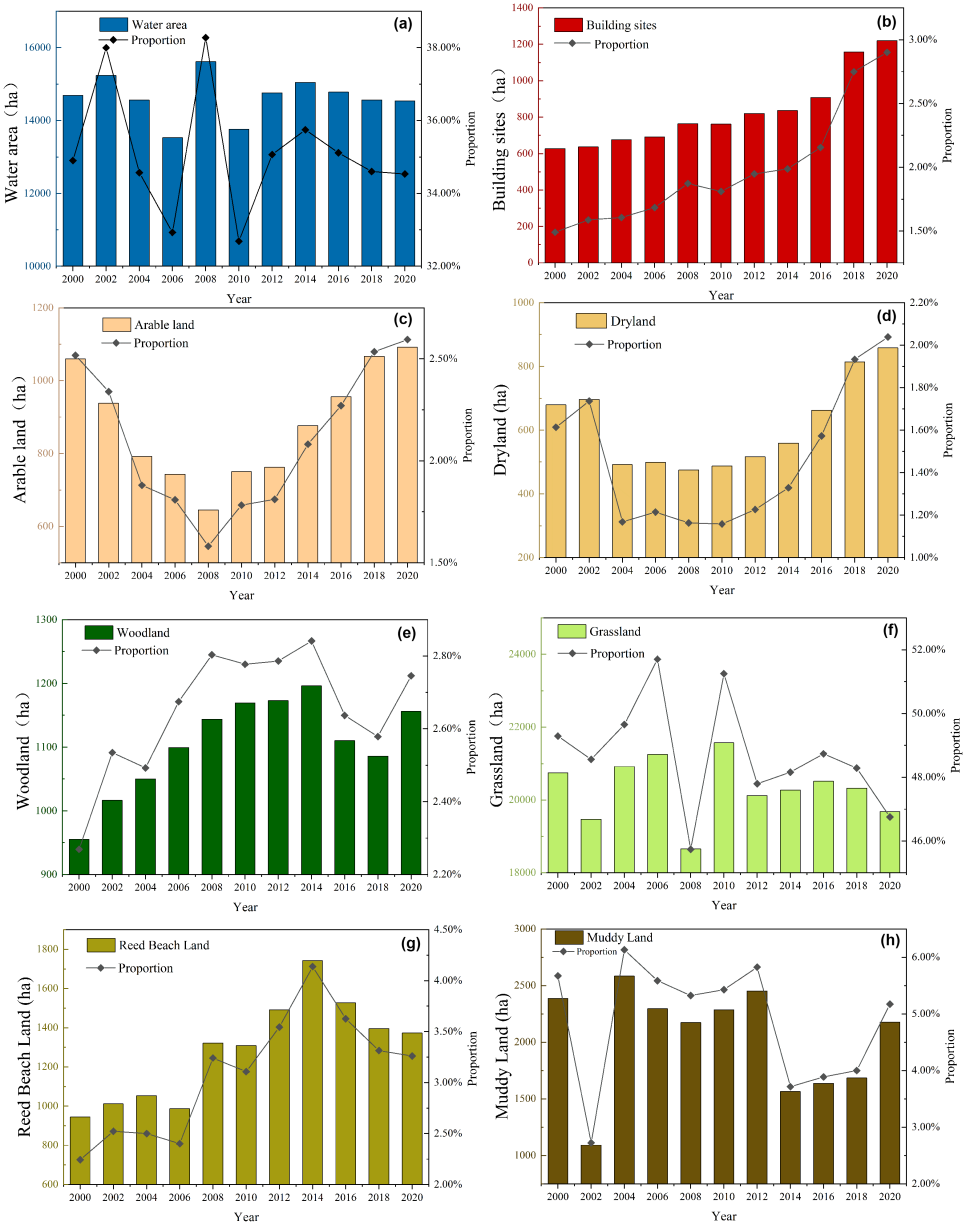
Land use change in the study area of Poyang Lake from 2000 to 2020. (a) Water area, (b) Building sites, (c) Arable land, (d) Dryland, (e) Woodland, (f) Grassland, (g) Reed Beach Land, and (h) Muddy Land.

The changes in land use types in the Poyang Lake wetland area in Jiangxi from 2000 to 2020 exhibited varying fluctuations (Figure 6). Based on these fluctuation patterns, we constructed land use transition matrices (Tables 6 and 7) to analyze the transitions between land use type in the Poyang Lake wetland. Additionally, the land use dynamic degree (Table 5) was used to indicate the speed of changes in different land use types.

**TABLE 5:** Land use dynamic attitude.

Type	Time		
	2000–2010	2010–2020	2000–2020
Water area	**−**0.006	0.006	**−**0.001
Building sites	0.021	0.060	0.047
Paddy field	**−**0.029	0.046	0.002
Dryland	**−**0.028	0.076	0.013
Woodland	0.022	**−**0.001	0.010
Grassland	0.004	**−**0.009	**−**0.003
Reedbeds	0.039	0.005	0.023
Mudflats	**−**0.004	**−**0.005	**−**0.004
Integrated dynamic attitude	0.004	0.005	0.003

The water area of Poyang Lake decreased from 34.9% in 2000 to 34.5% in 2020 (as shown in Figure 6a). Since 2000, the water area has been steadily declining, with occasional spikes in growth or decline. The overall trend indicates a constant decline, abrupt increase/decrease, and gradual stabilization.

The area occupied by building sites has continuously increased from 2000 to 2020 (Figure 6b). The fastest growth occurred between 2014 and 2020 when the area rose from 836.28 to 1221.08 ha, constituting 2.9% of the total land use.

The area of arable land exhibited a fluctuating pattern, transitioning through two stages (Figure 6c). In the first stage, the area continuously decreased from 1059.84 ha (2.51%) in 2000 to 645.21 ha (1.58%) in 2008. In the subsequent stage, there was a continuous increase from 2008 to 1092 ha (2.59%) in 2020.

Generally, the dryland region has an erratic increasing tendency, mostly as a result of population growth and rising demand for food and oil crops. The research area's dryland region makes up the tiniest portion, with a maximum contribution of less than 2%. It is mostly concentrated in the farming polders. Due to a deteriorating trend from 2002 to 2006, the largest sudden shift in dryland occurred in 2002 (Figure 6d). Additionally, from 2008 to 2020, the government strengthened forest land management, increased citizens' awareness of environmental protection, and introduced policies to restore farmland to forests and lakes (Ye et al., 2013). In 2020, the dryland area reached 858.15 hm ^2^, accounting for 2.03% of the total area.

In 2000, there were 955.26 ha of woodland, accounting for 2.26% of the watershed's area (Figure 6e). From 2000 to 2014, the woodland area increased yearly as a result of the protection of woodland in mountainous regions and the planting of forests (Zhang, Xu, & Xiao, 2020; Zhang, Zhou, & Li, 2020). After 2014, the demand for arable land and construction space increased. The forest area has been varied and altered, and the development of dams and flood control infrastructure has also had an influence on the woodland area's reduction due to floods (Feng et al., 2016).

The grassland area generally showed irregular changes, with large fluctuations from 2000 to 2010 and a stable area from 2010 to 2020 (Figure 6f) (Fan et al., 2018). The large fluctuation of the grassland area was mainly caused by the fact that Poyang Lake is a seasonal river, and most of the grassland is gathered around the water body of Poyang Lake.

The satellite image interpretation during the dry and abundant water periods suggests that in the dry period (Han et al., 2015), the grassland area accounts for a larger proportion due to the exposure of a large area of grassland at the bottom of the lake, while in the abundant period, the water body floods most of the grassland, reducing its proportion. In 2008 (Alireza et al., 2021), the water area accounted for a maximum of 38.28% of the whole period inundating most of the grass flats, resulting in a decrease in the area of grass flats.

From 2000 to 2020, the average annual percentage of mudflats was 4.86%, mainly in the shallow areas around lakes (Figure 6g).

The area of reed wetland exhibited a pattern of initial increase followed by a decrease. The analysis of reed wetland can be divided into two stages: the first stage, from 2000 to 2014, represented natural reed wetland changes, showing an overall upward trend. In 2000, the reed wetland area was 944.37 ha, constituting 2.2% of the total study area. From 2008 to 2014, this period was characterized by the coexistence of artificial planting and natural growth. During this time, the area of artificially planted reed continuously increased (Yang, Gao, et al., 2020; Yang, Xia, et al., 2020), leading to a rapid expansion of the reed wetland. By 2014, the reed wetland area peaked at 1742.11 ha, accounting for 4.1% of the total area. The second stage, from 2014 to 2020, marked a decline in reed wetland area. This decline was primarily attributed to the construction of bird‐watching platforms and migratory bird protection stations along the lakeside by various organizations. These constructions occupied significant reed wetland areas, leading to its shrinkage (Yang et al., 2018).

Changes in mudflat areas were primarily influenced by natural factors such as total annual precipitation and water level fluctuations. Human intervention in mudflats was relatively low, primarily related to water transport and sediment deposition (Figure 6h) (Mu et al., 2020).

In the 20 years from 2000 to 2020, the average annual proportion of grassland was 48.72%, the average annual proportion of water was 35.13%, the average annual proportion of mudflat was 4.86%, the average annual proportion of reed bed was 3.08%, the average annual proportion of forest land was 2.65%, the average annual proportion of paddy field was 2.11%, the average annual proportion of construction land was 1.98%, and the average annual proportion of dryland was 1.47%. The proportion of the area of the same period shows that the grassland > water > mud land > reed land > forest land > paddy land > construction land > dryland.

#### 3.2.2 Analysis of land use dynamic attitude and transfer matrix

As shown in previous studies, humans indirectly affect habitat quality through their effects on land use types (Gao et al., 2014; Ye et al., 2013). That is, the land type change is related to ecological quality. In this study, we analyze the characteristics of land use change in the area around Poyang Lake for over two decades, from 2000 to 2022, in terms of land use structure, dynamic attitude (Table 5), and type shift. We found the following characteristics.

#### 3.2.3 Land use change attitude

Table 5 shows that from 2000 to 2020, the paddy field increased by 0.002 points, indicating a minor and statistically insignificant rise in agricultural land area over the 20‐year period. Building land experienced the most significant change, exhibiting an overall increase of 0.047 points in two decades. Both grassland and watershed areas showed negative values, indicating a decrease in their respective areas.

#### 3.2.4 Land use transfer matrix

Changes in major land categories: (1) Transfer of building sites: From 2002 to 2012 (Table 6), the majority of building sites were transferred to waters, covering an area of 15.92 km^2^, which accounted for 92.93% of the building sites in 2012. The area transferred to other land types totaled 17.05 km^2^, making up 54% of the total Building site area.

**TABLE 6:** Land use transfer matrix from 2002 to 2012 (km^2^).

2002	2012							
	Arable land	Woodland	Shrubs	Grassland	Water area	Bare ground	Building sites	Total land
Arable land	11860.1	495.88	0	3.7	169.4	0.2	0.02	12529.3
Woodland	418.3	6394.5	0	0.6	3.0	0	0.03	6816.4
Shrubs	0.1	0.2	0.1	0.02	0	0	0.03	0.5
Grassland	2.8	0.2	0	7.6	4.6	4.4	0.04	19.6
Water area	692.6	24.3	0	1.4	3332.8	2.1	0.05	4053.3
Bare ground	0.4	0	0	1.7	4.0	8.5	0.07	14.8
Building sites	1.1	0	0	0.01	15.9	0	0.08	17.1
Total land	12975.3	6915.1	0.1	15.2	3529.8	15.2	0.32	23,451

From 2012 to 2022 (Table 7), construction land was predominantly transferred to cultivated land, spanning an area of 108.42 km^2^, equivalent to 8.58% of the total construction land area in 2022. The area transferred to other land types amounted to 289.30 km^2^, constituting for 22.90% of the total construction land area.

**TABLE 7:** Land use transfer matrix from 2012 to 2022 (km^2^).

2012	2022							
	Arable land	Woodland	Shrubs	Grassland	Water area	Bare ground	Building sites	Total land
Arable land	7568.3	1264.3	0.1	402.8	1325.8	14.6	2219.9	12795.7
Woodland	254.0	6336.4	0.1	96.5	86.8	6.0	104.1	6883.7
Shrubs	0	0.1	0	0.1	0	0	0	0.1
Grassland	2.1	0.41	0	4.9	3.0	2.3	2.4	15.1
Water area	88.9	2.71	0	37.3	3314.7	16.7	33.0	3493.3
Bare ground	0.2	0.11	0	6.3	3.0	5.3	0.2	15.1
Building sites	108.4	30.2	0	45.8	93.7	11.3	974.0	1263.3
Total land	8021.8	7634.1	0.2	593.6	4827.0	56.1	3333.6	24466.3

(2) Woodland transfer: Between 2002 and 2012 (Table 6), Woodland primarily transitioned to cultivated land, covering 418.26 km^2^, or 6.14% of the total Woodland area in 2012. The area transferred to other land types was 421.88 km^2^, accounting for 6.19% of the total Woodland area.

From 2012 to 2022 (Table 7), Woodland continued to shift mainly to cultivated land, covering 253.96 km^2^, which represented 3.69% of the total Woodland area in 2022. The area transferred to other land types was 547.35 km^2^, constituting 7.96% of the total Woodland area.

Changes in land use types are closely related to policies (Wang & Hu, 2012). For example, the continuous increase in forest land until 2014 (Figure 6) and the significant expansion of construction land have been influenced by relevant policies (http://drc.jiangxi.gov.cn/). According to the role of urbanization: 2000–2005, the counties and districts in the Poyang Lake area were in a primary development stage of urbanization with a slow development rate (Michishita et al., 2012). The arable land and other land types, such as unused land, were converted into construction land on a large scale (Table 7).

The reasons for the decrease in arable land were twofold (Liu & Zhu, 2010): On the one hand, due to the imbalance of socioeconomic development, many young people went out to work, resulting in the conversion of arable land to grassland or unused land; on the other hand, due to the continuous urbanization, the sharp expansion of development and construction projects and the increasing occupation of arable and forest land by other nonagricultural projects resulted in the shrinkage of arable and forest land (Vallecillo et al., 2016).

### 3.3 Driving force analysis

#### 3.3.1 Factor detection results and analysis

**FIGURE 7:**
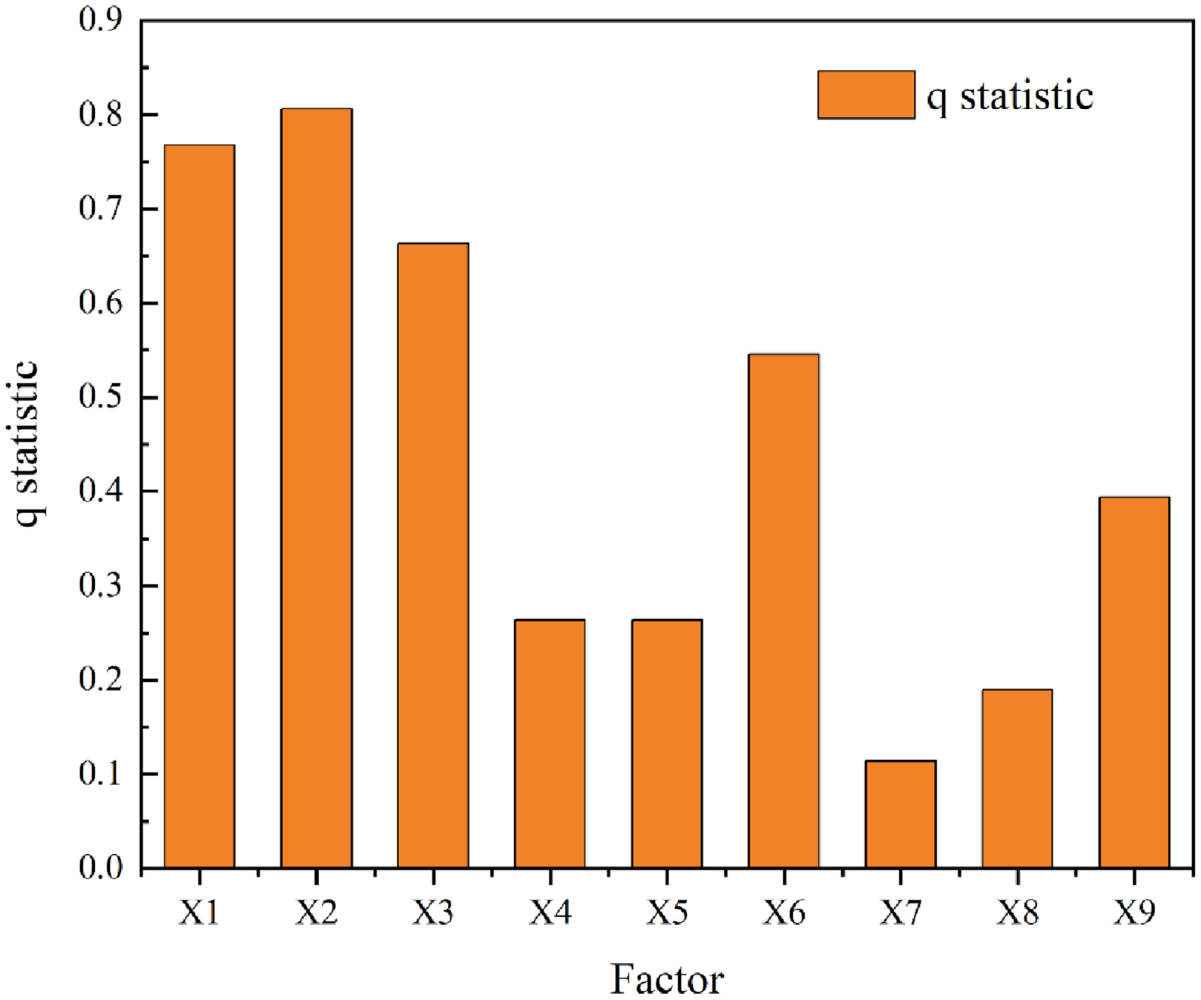
Results of habitat quality change factor detection in the county around Poyang Lake. (X1: Elevation, X2: Slope, X3: Slope direction, X4: Precipitation, X5: Temperature, X6: Population density, X7: Total population at the end of the year, X8: Gross Domestic Product, and X9: Area of the district).

The factor probing findings demonstrate that the factors X1 (elevation), X2 (slope), X3 (slope direction), and X6 (population density) have substantial explanatory power and have q‐values greater than 0.40 (Figure 7).

The population density and total population at the end of the year can provide an overview of the demographic characteristics and regional population distribution; the q‐values of these variables, which affect the spatial pattern of habitat quality change, are 0.545437 and 0.114285, respectively. The indicator of the level of regional economic development is the gross domestic product. In 2020, the q‐value in the Poyang Lake County area reached 0.189686. The primary factors affecting habitat quality change are the population density and economic development level. The natural geography elements X1 (elevation), X2 (slope), and X3 (slope direction) all have substantial explanatory degrees (over 0.66) for habitat quality change. The habitat quality shift is best explained by the socioeconomic variables X6 (population density) and X9 (administrative area). For understanding changes in habitat quality, the natural geography element is more important than the socioeconomic factor.

#### 3.3.2 Interaction detection results and analysis

**FIGURE 8:**
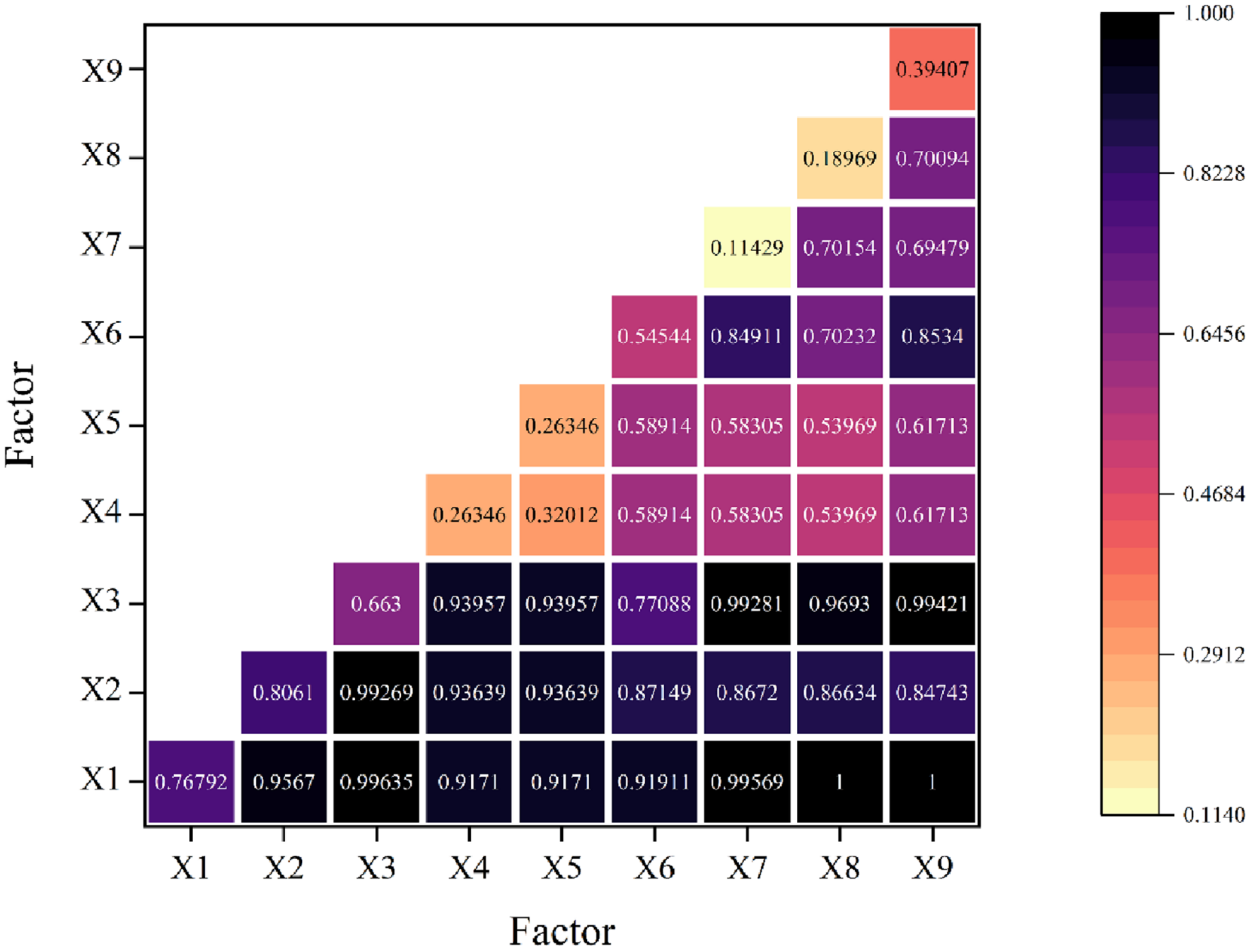
Interaction detection results of habitat quality change factors around Poyang Lake. (X1: Elevation, X2: Slope, X3: Slope direction, X4: Precipitation, X5: Temperature, X6: Population density, X7: Total population at the end of the year, X8: Gross domestic product, and X9: Area of the district).

In the counties near Poyang Lake, the factors influencing habitat quality change varied. The findings of interaction detection demonstrate that interactions between factors have a larger explanatory power on habitat quality change than a single component (Figure 8).

The explanatory degree of interaction between X6 (population density) and X7 (total population at the end of the year), excluding the nonnatural components, is 0.8491. Population density (X6) and administrative district area (X9) interact with an explanatory degree of 0.8534. The statistics show that the habitat quality change is mostly driven by population density, total population at the end of the year, and administrative district area as the common driving variables, which are more significant than the level of explanation for each individual element.

#### 3.3.3 Ecological sounding results and analysis

**FIGURE 9:**
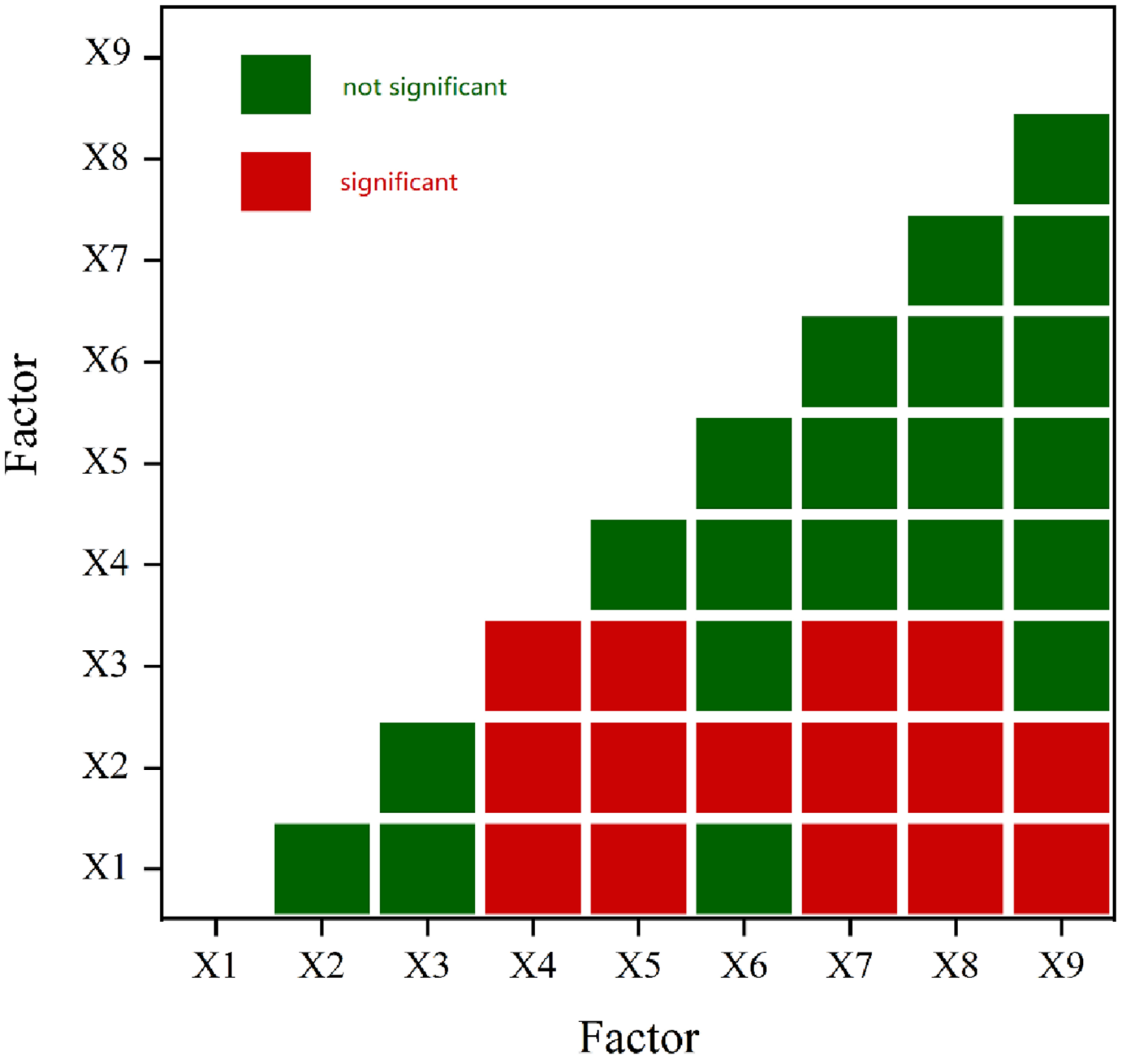
Ecological survey results around Poyang Lake. (X1: Elevation, X2: Slope, X3: Slope direction, X4: Precipitation, X5: Temperature, X6: Population density, X7: Total population at the end of the year, X8: Gross domestic product, and X9: Area of the district).

Ecological probing is used to assess the relationship between two factors for variations in the dependent variables, that is, to determine if there are variations in the impacts of soil type and elevation on the dependent variables in the counties around Poyang Lake (Figure 9). There are variations in the effects of precipitation, elevation, slope, and slope direction, as well as in the effects of population density and slope orientation, on habitat quality change. Additionally, there are variances in the height, slope, and aspect of habitat quality change, as well as in the total population, GDP, and year‐end GDP. Administrative area, elevation, and slope orientation all have different effects on habitat quality change. There are no appreciable variations in how other factors affect habitat quality change.

In summary, the following points can be highlighted: ① The influence of three factors, namely elevation, slope, and slope direction, on the spatial pattern of habitat quality change is stable. ② Nonnatural factors: population density, total population at the end of the year, gross domestic product, and administrative district area do not differ significantly in their influence on the spatial pattern of habitat quality change.

## 4 DISCUSSION

### 4.1 Human activities put pressure on habitat quality

In this study, the InVEST model was used to analyze the effects of human activities on habitat quality and the level of habitat degradation. The results showed that the level of habitat quality was lowest in areas where human activities were concentrated, such as cities (Figure 2). This phenomenon is evident in cities with concentrated population, such as Nanchang and Jiujiang, where the habitat quality of construction land is below 0.1; on the other hand, the habitat quality of sites with less human activity is high, such as forest land, where the habitat quality level is above 0.8, and cultivated land, as a buffer zone between forest land and construction land, where the habitat quality is around 0.5. The habitat quality can also be used as an indicator to measure the ecological environment and the impact of human activities, that is, forest land that is not fully subjected or weakly affected by human activities, arable land that is affected to some extent, and construction land that is fully affected by human activities (Lei et al., 2022; Tang et al., 2022; Wang, Ma, et al., 2022; Wang, Ye, et al., 2022).

Habitat deterioration is evident in the city of Nanchang, which is at the center of the Poyang Lake basin, and the extent of degradation increases as one approaches the built‐up region. Rivers, lakes, and mountains tend to have low levels of habitat degradation, and habitat quality in these regions is excellent due to the lesser impact of human activities (Figure 2).

Human activities affect habitat quality mainly through population density, total population at the end of the year, and the size of administrative areas (Figures 3 and 5). Additionally, geographical patterns in regional habitat quality are influenced by the interaction of natural and socioeconomic variables (Sun et al., 2019; Zhang, Xu, & Xiao, 2020; Zhang, Zhou, & Li, 2020). According to Zhao et al. (2018), socioeconomic variables such as population density, which are directly tied to human activities, pose a danger to habitat quality and can result in habitat loss and degradation. Nanchang, the central city of the Poyang Lake basin, is a good example of this.

Land use change can have significant ecological impacts, and it is closely related to human activities. One of the main drivers of land use change in the wetlands around Poyang Lake is population growth. Urban expansion in the Poyang Lake basin from 2000 to 2020 is closely related to population increase, and policies have significant impacts on land use (Zhen et al., 2011), especially after the 1998 flood. In the middle and lower reaches of the Yangtze River Basin, China began to put wetland restoration strategies into practice. Among all land use types, the impact of built‐up land in towns and cities on habitat quality is particularly evident. Threatened habitat quality includes both built‐up land and cultivated land for agriculture. Liu and Zhu (2010) suggest that population expansion has a significant impact on changes in carbon emissions in the ecological urban agglomeration around Poyang Lake. This may exacerbate the harm to the wetlands of Poyang Lake. Predatory use of land by humans degrades natural resources and the environment, affecting their sustainable utilization (Shi et al., 2023).

This study evaluates the factors driving changes in habitat quality within the context of land use and geoprobes at Poyang Lake. However, the urban land area continues to expand. Addressing the challenge of rational planning within the current situation of the Poyang Lake watershed requires careful attention.

### 4.2 Limitations of the study

However, this study has limitations due to the lack of appropriate data and information. Primarily, our discussion in this study focused on the habitat quality of Poyang Lake in 2020, with limited research available on long‐term habitat quality trends. Additionally, the correlation between habitat quality levels and population, as well as other factors, was not comprehensively quantitatively studied in this research. Therefore, further studies are needed to address these limitations and enhance the overall adequacy of the analysis.

## 5 CONCLUSIONS

Poyang Lake, as a representative of freshwater lake wetland, holds immense significance for migratory bird conservation and wetland management in China. However, its ecological environment has gradually weakened in recent years due to intensified human activities. Changes in various land use types within the watershed directly impact its habitat quality. Despite this, there have been fewer studies on the drivers of habitat quality conditions in Poyang Lake. In this study, we quantitatively evaluated the drivers of habitat quality in Poyang Lake using the InVEST model and Geodetector. Additionally, we interpreted the results in the context of land use changes from 2000 to 2020. The findings revealed the following.

The increase in urbanization significantly affected habitat quality. Low and high value areas of habitat quality were concentrated in the forests of towns and principle towns, respectively. Anthropogenic activities were primarily driven by population density, the total number of people at the end of the year, and the area of administrative regions. The land allocated for construction continuously increased from 2000 to 2020, highlighting the need for effective policy measures to enhance habitat quality of the Poyang Lake Basin and to restrict further intensification of anthropogenic activities. Moreover, the stressor parameters utilized in this study can serve as valuable references for similar watershed environments. InVEST stressor variables from similar environments can also be applied to other watersheds facing comparable anthropogenic challenges, even in cases where specific data are lacking.

## AUTHOR CONTRIBUTIONS

**Wenrui Yuan:** Conceptualization (supporting); methodology (lead); writing – original draft (lead). **Lingkang Chen:** Conceptualization (lead); funding acquisition (lead); supervision (lead); writing – review and editing (lead). **Haixia Chen:** Methodology (equal); writing – review and editing (supporting). **Shaofu Deng:** Data curation (supporting); software (supporting); visualization (lead). **Hong Ji:** Resources (supporting); supervision (lead). **Fenshuo Liang:** Software (supporting).

## CONFLICT OF INTEREST STATEMENT

The authors declare that they have no known competing financial interests or personal relationships that could have appeared to influence the work reported in this article.

### OPEN RESEARCH BADGES

This article has earned an Open Data badge for making publicly available the digitally‐shareable data necessary to reproduce the reported results. The data is available at [https://datadryad.org/stash/dataset/doi:10.5061/dryad.7wm37pvzh].

## Data Availability

Data from the results of this study are freely available in https://datadryad.org/stash/dataset/doi:10.5061/dryad.7wm37pvzh. Private access to download the data files URL: https://datadryad.org/stash/share/U8DLConxzfikAIRI3nfPzpcRpwNnA8obEjW2Ga8DwUk, reference number 58.
